# Hydrocele and Spermatocele, with Report of Cases

**Published:** 1908-05

**Authors:** W. L. Champion

**Affiliations:** Atlanta, Georgia


### HYDROCELE AND SPERMATOCELE, WITH REPORT OF CASES.*

BY W. L. CHAMPION, M. D., ATLANTA, GEORGIA.

Hydrocele, or an accumulation of fluid in the tunica vaginalis, being a condition we are so frequently called upon to relieve, so easily recognized, and as a rule, so successfully cured by the injection of carbolic acid, I desire to report fifty-one cases taken from my record book; and also to include, on account of its rarity, three cases of spermatocele.

When there is an accumulation of clear serous fluid in the tunica vaginalis, it is termed hydrocele; when the fluid contains blood, hematocele; and when spermatozoa are present, spermatocele. There are several varieties of hydrocele: acute, chronic, multilocular, congenital, infantile, inguinal, and hydrocele of the

*Read before Medical Association of Georgia, Fitzgerald, April 15, 16, 17, '08

spermatic cord. In this short article the various kinds will not be described,--only dividing hydrocele into two classes: symptomatic and idiopathic.

Symptomatic hydrocele follows a diseased testicle; idiopathic hydrocele develops without any known cause, or, not knowing the cause, this name is applied to the condition.

In the report to follow, ten of the fifty-one cases did not have any disease of the testicle or epididymis, nor did they give a history of gonorrheoa or syphilis. The fact that so few males escape venereal diseases, and the possibility of injury being the cause, all hydrocele might be classed as symptomatic. With the patient in a dark room and a light behind the tumor, diagnosis is easily made if the fluid is clear; if not translucent and eliminating hernia, a hypodermic needle inserted will clear up the diagnosis; unless the hydrocele is very large, the testicle can be felt to determine whether it is sensitive, hard, enlarged, or nodular. As a rule when a testicle is syphilitic there is fluid in the tunica vaginalis. In small hydroceles incision, aspiration or tapping will occasionally perfect a cure, but in large tumors after tapping without injection, the fluid will gradually accumulate.

Casper, in his text-book on genito-urinary diseases, states that the procedure of tapping and injecting irritating substances, such as tincture of iodine and carbolic acid, is not absolutely certain nor without danger; therefore, he favors the more radical operation by means of open incision.

Green and Brooks, in their recent work on diseases of the genito-urinary organs, state: "It has been a common custom for a great many years to inject into the sac through the trocar, a few drops of a powerful destructive agent, with the object of setting up an adhesive inflammation between the walls of the tunica that will cause them to adhere and thus prevent the reformation of fluid. This method is sometimes successful, but personally the writers prefer one of the radical operations,—that is incision." I have never seen any bad results nor toxic effects from the use of carbolic acid, and have injected a dram of the acid in a hydrocele sac. Keyes, in late edition on genito-urinary diseases, says: "After using carbolic acid injections in many cases ranging in age from two months to eighty years, in no case has any complication or serious reaction occurred in his hands."

I do the operation in the office, first cocainizing the part to be punctured, which makes the operation painless, with the exception of the first effect of acid, which is a severe smarting pain lasting perhaps for a minute. Before introducing the trocar, the testicle should be located, so as to avoid injuring it. Some operators after emptying the sac through the canular, inject the acid with a hypodermic syringe, inserting the needle at a location distant from the opening made to withdraw the fluid. The needle is inserted before the fluid is drawn off; after the fluid escapes through the canular, the syringe containing the acid is attached to the needle and acid injected.

I prefer to empty the sac and inject twenty to thirty minims of the pure acid through the canular in the position it is in when the fluid is drawn, connecting the syringe with the canular, or preferably, using a needle a fraction longer than the canular, and passing it through the canular to the bottom of the sac, thus preventing the acid from coming in contact with the skin or escaping into the tissues. Before injecting the acid, care should be taken to remove all the fluid, as a small quantity left in the sac may cause the operation to result in failure. Immediately after injecting the acid, remove the canular and knead the scrotum so as to distribute the acid over the surface of the sac. Close the puncture with collodion, and request the patient to remain at home for twenty-four hours, as there will be some soreness and swelling due to reaction. A suspensory bandage should be worn until the scrotum resumes its natural size.

I believe the operation will always result in a cure without any complications if aseptically done, except in cases where the walls of the sac are very much thickened or the accumulated fluid results from syphilis or tuberculosis of the testicle.

Of the fifty-one cases here reported, twenty-eight had gonorrhoea, or gave a history of the disease; ten gave no history of gonorrhoea or syphilis; eight had syphilitic involvement of the testicle; four were tubercular; and one cancerous. Three of the cases were taped and never reported again. In three of the syphilitic cases injected, the fluid returned and they were injected again after being on anti-syphilitic treatment for a few months. After tapping a hydrocele, if the testicle is found to be enlarged, hard and irregular in shape, indicating syphilitic or tubercular condition, it is useless to inject carbolic acid. The patient should be put on proper treatment for such condition, and if the testicle is syphilitic, the hydrocele as a rule will disappear as the testicle becomes normal.

It is rare to find a cyst springing from the testicle or fluid in the tunica vaginalis containing spermatozoa. This condition is termed spermatocele, and is produced by inflammation of the seminiferous tubules, thus causing retention of the semen in the constricted tube forming cyst. Should the cyst rupture into the tunica vaginalis, the condition resembles hydrocele. On account of its rarity, without giving a detailed report, I mention three cases that have come under my observation. Two of the cases consulted me account of an enlargement of the testicle. The first case in 1904, and the other in 1907. Both cyst were small, containing five or six drams of fluid alive with spermatozoa, and were cured by incision. The third case was seen by request of Dr. L. A. Fowler, at the Federal Prison. About two ounces of fluid of a milky appearance was drawn off, which under the microscope showed the field swarming with spermatozoa.

313 and 314 Prudential Building.

References:

*Surgical Diseases of the Genito-Urinary Organs*, E. L. *Keys*. 1903.

*A Text-Book of Genito-Urinary Diseases, Dr. Leopold Casper*, 1906.

